# Effect of Centrifugation Methods on the Regenerative Potential of Concentrated Platelet-Rich Fibrin: An *In Vitro* Study

**DOI:** 10.1055/s-0045-1812112

**Published:** 2025-11-07

**Authors:** Kwartarini Murdiastuti, Abyan Teguh Saputro, Dharmmesti Anindita Wijayanti

**Affiliations:** 1 Periodontics Specialisation Study Program, Faculty of Dentistry, Universitas Gadjah Mada, Yogyakarta, Indonesia; 2 Department of Periodontics, Faculty of Dentistry, Universitas Gadjah Mada, Yogyakarta, Indonesia

**Keywords:** horizontal centrifugation, fixed-angle centrifugation, concentrated platelet-rich fibrin, TGF-β1, PDGF-AB, proliferation, migration

## Abstract

**Objective:**

A new type of platelet concentrate used in regenerative periodontal therapy, called concentrated platelet-rich fibrin (C-PRF), contains more growth factors than previous generations of platelet concentrate. Compared to fixed-angle centrifugation, a recently discovered technique called horizontal centrifugation can produce C-PRF with a more evenly distributed quantity of platelets and improved biological properties. This study aims to determine the effect of horizontal and fixed-angle centrifugation methods on the release of transforming growth factor β1 (TGF-β1) and platelet-derived growth factor AB (PDGF-AB), as well as on the migration and proliferation of osteoblast cells.

**Materials and Methods:**

This study used two test groups: a horizontal group and a fixed-angle group. The release of growth factors TGF-β1 and PDGF-AB was measured at five time points: the first, third, seventh, 10 ^th^ , and 14th days of incubation, using the enzyme-linked immunosorbent assay. To determine the effect of C-PRF type on osteoblast cell migration, a scratch wound healing assay was conducted 24 hours after incubation. Osteoblast cell proliferation was counted using a 450-nm microplate reader to determine the number of cells in the well plate on the first, third, and fifth days, with the Cell Counting Kit-8. The data were examined using two-way ANOVA (analysis of variance) and post hoc LSD (least significant difference).

**Results:**

The findings indicated a statistically significant increase in the release of TGF-β1 and PDGF-AB in the horizontal group compared to the fixed-angle group over a 14-day period. The horizontal group's TGF-β1 release increased by a factor of 1.5 compared to the fixed-angle group, peaking on day 7. Following a 24-hour incubation period and microscopic examination, the horizontal group exhibited greater osteoblast migratory activity than the control group and the fixed-angle centrifugation group. Horizontal centrifugation C-PRF had a significantly greater effect on osteoblast proliferation than fixed-angle centrifugation. The horizontal group's proliferation rate surpassed that of the fixed-angle group by up to 1.5 times on the third day of observation.

**Conclusion:**

Compared to fixed-angle centrifugation, the horizontal method of preparing C-PRF has a greater effect on the release of growth factors and the behavior of osteoblasts.

## Introduction

In regenerative periodontal therapy, tissue engineering aims to generate new alveolar bone, cementum, and periodontal ligament attachment to surrounding tooth tissue. The primary elements of tissue engineering are bone grafts, biological materials, and membranes. Biological materials are expected to enhance both the initial wound healing phase and overall tissue regeneration by serving as a reservoir of cytokines, growth factors, and human protein products. 1 Platelet concentrate is a common type of biological material. 2

Platelet-rich plasma (PRP) is a first-generation platelet concentrate that is widely used in regenerative procedures for periodontal treatment. Despite the success of PRP, concerns were raised over the autologous properties of the procedure involving an anticoagulant that may affect its regenerative potential. 3 Therefore, platelet-rich fibrin (PRF) was developed by removing the anticoagulating agent. Centrifugation protocols operating at higher rpm/RCF (relative centrifugal force) values produce solid fibrin clot matrices rich in leukocytes, known as leukocyte and PRF (L-PRF) membranes. However, further evidence showed that the majority of platelets and leukocytes were pushed out of the buffy coat layer due to the high centrifugal force and into the red blood cell layer at the bottom of the tube, thus excluding them from the PRF membrane. To overcome this limitation, pioneering research on the low-centrifugation concept was conducted. This demonstrated a greater concentration of platelets and leukocytes in the upper layer of PRF after reducing the duration and speed of centrifugation. 4

This optimization increased the release of growth factors and enhanced the regenerative potential of the PRF. This protocol development also revealed that, with a 3 to 5 minute spin cycle at a low *g* -force, the plasma will remain in a liquid state for 15 to 20 minutes before coagulation occurs. This liquid PRF, known as injectable PRF (i-PRF), can be extracted and injected directly into tissues. It offers better handling properties and clinical applications than L-PRF. 5 Further findings revealed that a higher centrifugation protocol (2,000 RCF for 8 minutes) produced concentrated PRF (C-PRF), demonstrating a 10-fold increase in platelet and leukocyte concentration. This is thought to be due to the larger disparity between the speed of the centrifuge and the horizontal instruments, which are more effective at distributing platelets, leukocytes, and growth factors across the blood layer and at segregating cell types according to density. 6 A previous study demonstrated that C-PRF had the highest concentrations of platelets and leukocytes compared to L-PRF exudate and the combination of the two. An experiment on interdental papilla tissue regeneration in male Sprague-Dawley rats concluded that C-PRF stimulated vascularization more effectively than the other PRF groups. 7

The fixed-angle centrifugation method has been used to produce platelet concentrates for many years. Recently, the horizontal centrifugation approach has been used to manufacture PRFs. 8 Because it applies more uniform pressure to the blood-filled tube throughout the screening procedure, this approach is being studied as a means of achieving a more even distribution of leukocytes and platelets on the PRF membrane. This method produces PRF with improved regenerative potential, known as horizontal PRF. 8 9

Previous studies have shown that PRF may affect the release activity of growth factors, as well as cell migration and proliferation, in the process of wound healing. 9 10 11 A review of these studies concluded that, in addition to a higher neutrophil count, A-PRF significantly increased the release of growth factors, such as transforming growth factor β1 (TGF-β), platelet-derived growth factor (PDGF), and vascular endothelial growth factor (VEGF), within 10 days. 12 The migration speed of human dental pulp stromal cells was observed to be at its highest after being cultured in 10% A-PRF for 24 hours compared to the DMEM (Dulbecco's Modified Eagle Medium) control group. 13 Wang et al found that the group receiving PRF showed an increase in osteoblast cell proliferation starting on the third day and peaking on the fifth day. 10 A similar study comparing C-PRF with the earlier generation of PRF, i-PRF, was carried out by Fujioka-Kobayashi et al. 11 This study found that the C-PRF group showed increased cell proliferation activity on the third and fifth days, with a gradual rise in growth factor release over the 10-day observation period. 11

## Materials and Methods

### Fixed-Angle and Horizontal C-PRF Preparation

All research procedures involving human beings have passed the institutional ethical feasibility assessment with code 106/UN1/KEP/FKG-RSGM/EC/2024. Blood samples were collected from three volunteers who consented to having their blood used as study material. Donors were selected based on the following inclusion criteria: (1) aged between 18 and 60; (2) platelet count >150,000 mc/L; and (3) hemoglobin >12 g/dL.

**Fig. 1:**
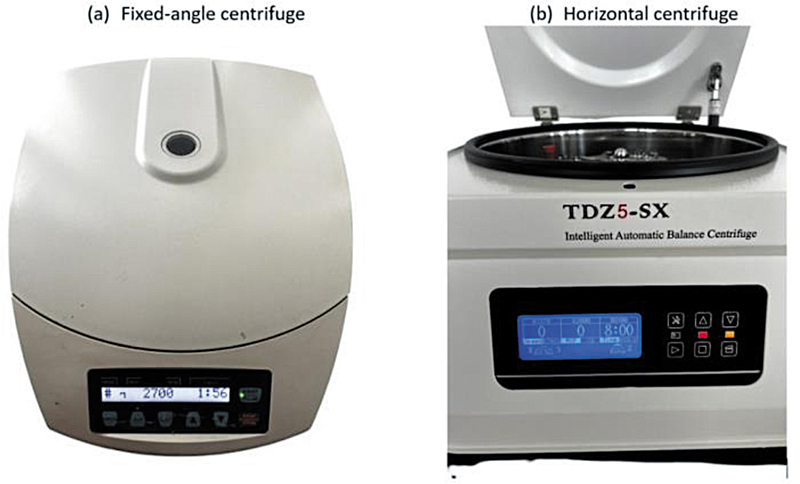
The two types of centrifuge used in this study, **(a)** fixed-angle centrifuge and **(b)** horizontal centrifuge.

Six PET tubes containing 10 mL of blood (BD Vacutainer, United States) were divided into two groups and centrifuged at 2,000 × *g* RCF for 8 minutes using both a fixed-angle centrifuge (EBA 280, Hettich, Germany) and a horizontal centrifuge (Hunan Biojoint, China; Fig. 1 ). Following preparation, the harvesting procedures adhered to the C-PRF optimization protocol from an earlier study by Miron et al. 6 A 1 mL layer of liquid, which separated the plasma and red blood cell corpuscles and was located on top of the buffy coat, was harvested from each tube individually using a syringe with an 18G needle (Terumo, Japan). Once identified, the C-PRF sample was placed in a six-well culture plate (Iwaki, Japan) containing 5 mL of cell culture medium (Gibco, Thermo Fisher Scientific, United States) and processed and observed as described below.

### Growth Factor Release Quantification

Samples were incubated in a CO _2_ incubator at 37 °C (Binder, Germany) to allow growth factors to be released into the cell culture medium. This was done in order to determine the concentrations of growth factors released by horizontal and fixed-angle C-PRF on days 1, 3, 7, 10, and 14. Five milliliters of culture medium were obtained at each observation period and stored at −80°C in a freezer (New Brunswick Innova U535, Eppendorf, Germany). Five milliliters of fresh culture medium were added to the well plate. This process was repeated until the final day of observation. Following the manufacturer's instructions, quantification was performed using TGF-β1 (detection range = 31.2–2,000 pg/mL, RK00055, ABclonal Technology, United States) and PDGF-AB (detection range = 78.13–5,000 pg/mL, ELK1089, ELK Biotechnology, United States). The absorbance value was determined at 450 nm using a microplate reader (iMark, Bio-Rad, United States).

### Osteoblast Cell Culture

A stock of 100 μL of MG-63 osteoblast cells was placed in a centrifugation tube filled with 10 mL of phosphate-buffered saline, and the osteoblast cells were extracted. The tube was then centrifuged for 10 minutes at 24 °C and 2,000 rpm. The pellet and the supernatant are the two components resulting from the centrifugation process. The pellets were then homogenized by dissolving them once more in the culture medium. After transferring the solution to a petri dish, the dish is filled with the culture medium until it reaches a volume of 7 mL. The petri dishes are then incubated for 24 hours at 37°C with 5% CO _2_ . Osteoblasts can be extracted once the cells have proliferated to reach confluence. 14

### Osteoblast Cell Migration

**Fig. 2:**
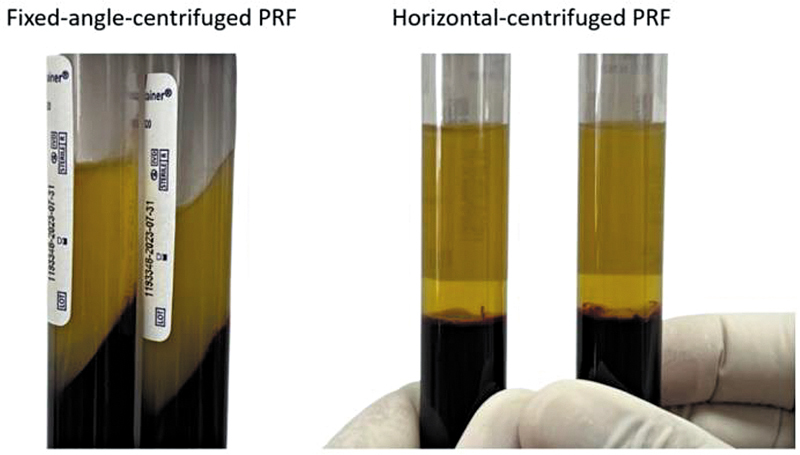
Visual documentation of C-PRF after centrifugation with an RCF at 2,000 g for 8 minutes in a fixed-angle centrifuge and a horizontal centrifuge. C-PRF, concentrated platelet-rich fibrin; RCF, relative centrifugal force.

The osteoblast cells were immersed in the control medium and horizontal and fixed-angle C-PRF in a six-well plate and then incubated for 24 hours. The scratch wound healing assay method was used to observe osteoblast cell migration under a microscope, with the percentage of area closure by osteoblast cells being measured ( Fig. 2 ). 15

### Osteoblast Cell Proliferation

Culture media with a cell density of 2.5 × 10 ^3^ were placed in a 96-well plate and cultured at 37°C in an incubator with 5% CO _2_ . Observations were made after 1, 3, and 5 days of incubation. At each time point, 10 μL of Cell Counting Kit-8 solution (Abbkine, China) was added to each well. The plate was then incubated for an additional 2 hours at 37 °C in an incubator with 5% CO _2_ . Osteoblast cell counts were determined using a microplate reader set to 450 nm. 10

### Statistical Analysis

All experiments were performed in triplicate. The means and standard errors were calculated and the data obtained in this study were analyzed statistically using a two-way ANOVA (analysis of variance) and an LSD (least significant difference) post hoc test in IBM SPSS Statistics.

## Results

### Growth Factor Release

**Fig. 3:**
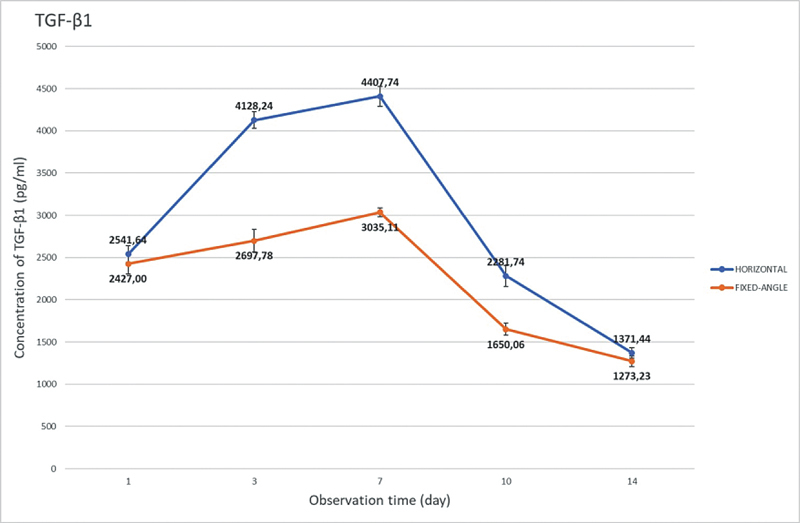
TGF-β1 concentration at each observation time point over 14 days in horizontal and fixed-angle C-PRF groups. C-PRF, concentrated platelet-rich fibrin.

This study measured the concentration of released growth factors (TGF-β1 and PDGF-AB) using ELISA (enzyme-linked-immunosorbent assay) over a period of 14 days. According to the data shown in Fig. 3 , there was a statistically significant difference in the release of TGF-β1 and PDGF-AB between the horizontal C-PRF group and the fixed-angle C-PRF group. TGF-β1 release was similar in both groups on the first day of observation, then gradually increased on day 3, reaching a peak on day 7, with the horizontal group's concentration increasing 1.5 times more (4,408 pg/mL) than the fixed-angle group's (3035 pg/mL). After that, it gradually decreased on days 10 and 14.

**Fig. 4:**
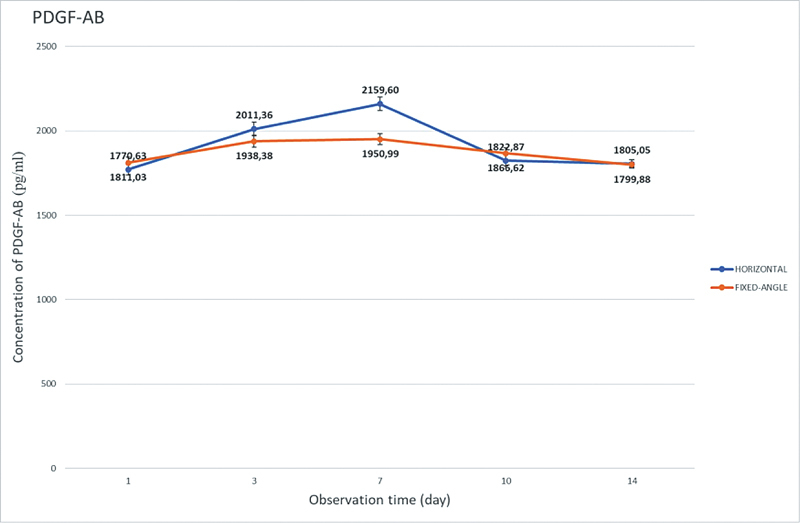
PDGF-AB concentration at each observation time points over 14 days in horizontal and fixed-angle C-PRF groups. C-PRF, concentrated platelet-rich fibrin; TGF-β1, transforming growth factor-β1.

The release of PDGF-AB in both groups followed a similar pattern to that of TGF-β1. Horizontal groups were statistically significant compared to fixed-angle groups at each observation time point, as shown in Fig. 4 .

### Osteoblast Cell Migration

**Fig. 5:**
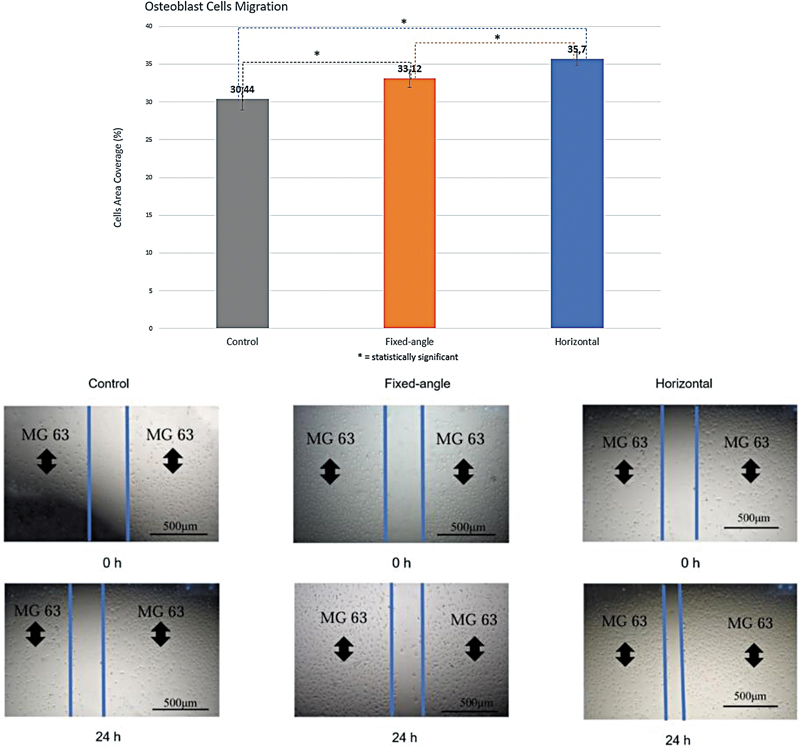
Migration activity of osteoblast after 24 hours of incubation.

Following 24 hours of incubation and observation under a microscope, the horizontal C-PRF group exhibited the highest percentage of area coverage by osteoblasts (35.7%). Fig. 5 shows that osteoblast migration activity was higher in the horizontal C-PRF group than in the control group (30.44%) and the fixed-angle C-PRF group (32.38%).

### Osteoblast Cell Proliferation

**Fig. 6:**
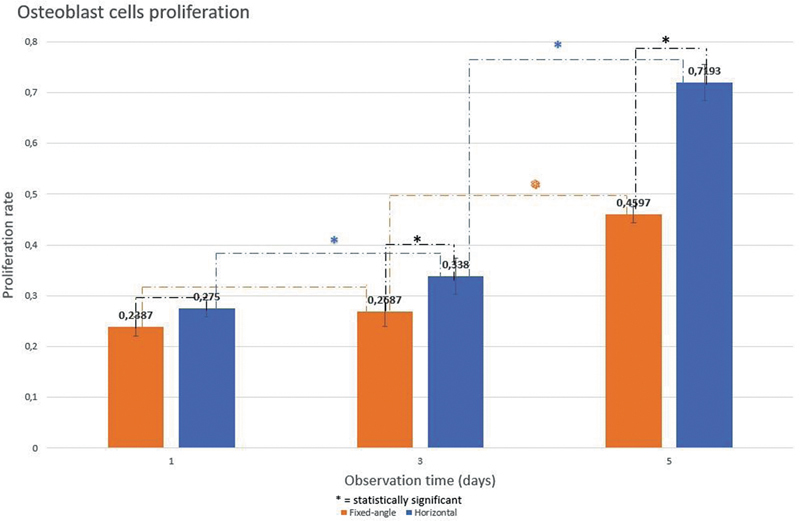
Proliferation rate of osteoblast from the C-PRF horizontal group and fixed-angle group on each observation day. C-PRF, concentrated platelet-rich fibrin.

Compared to the fixed-angle centrifugation group, the C-PRF horizontal centrifugation group indicates a higher osteoblast proliferation rate. As shown in Fig. 6 , on the third day of the observation period, the growth of the horizontal group (0.7193) was up to 1.5 times higher than that of the fixed-angle group (0.4597).

## Discussion

The release pattern of the growth factors TGF-β1 and PDGF-AB exhibited by C-PRF in this study is consistent with previous findings, which showed cellular activity and growth factor release over a period of 10 days, after which it began to diminish. 16 Other studies reveal that, in both liquid and solid PRF, five types of growth factor (including TGF-β1) exhibit an increasing release pattern starting from the first hour after incubation, peaking on day 7, and then declining on day 10. 17 The production of TGF-β1 and PDGF-AB in both groups may be related to the physiological state of the dominant cell responsible for generating growth factors in C-PRF, specifically, platelets and leukocytes.

Growth factors, such as TGF-β1 and PDGF-AB, are produced by activated platelets and leukocytes. The number, distribution, and intensity of activated cells in PRF affect growth factor release. An increase in the number of platelets and leukocytes is proportional to growth factor release. 18 19 The angulation of the tube during horizontal centrifugation prevents cells from being forced against the tube's distal wall, thereby limiting the possibility of them crashing into the tube and becoming damaged. Horizontal centrifugation decreases the likelihood of cell damage by separating the blood cells from the plasma in the middle of the tube rather than pushing them towards the wall of the tube. Horizontal centrifugation results in a more uniform distribution of cells in the upper layer than fixed-angle centrifugation, which concentrates cells at the distal wall. 9 Miron et al conducted a study analyzing cell blood counts to quantify cell numbers, and found that horizontal centrifugation significantly increased platelet and leukocyte numbers and concentrations compared to fixed-angle centrifugation. 6 Horizontal centrifugation resulted in a more uniform distribution of platelets and leukocytes across PRF matrices compared to fixed-angle centrifugation. 20

The results of this study showed that the C-PRF from the horizontal centrifugation group had a higher concentration of both TGF-β1 and PDGF-AB than the fixed-angle centrifugation group at all observation times. These findings are consistent with those of previous studies. Investigations into the effect of rotor angle (fixed-angle or horizontal) on growth factor release have reported that horizontal centrifugation leads to a twofold increase in the release of PDGF, VEGF, and fibroblast growth factor 2 compared to fixed-angle centrifugation. 21 Another study by Gheno et al also exhibited a significant increase in PDGF and VEGF release from PRF produced by horizontal centrifugation, with a concentration twice that of PRF produced by fixed-angle centrifugation. 22 In contrast to these findings, Al-Maawi et al found no statistically significant difference between horizontal and fixed-angle centrifugation protocols in terms of the release of TGF-β1 and VEGF from liquid PRF matrices over 7 days of observation. 23

C-PRF releases a higher concentration of growth factors, particularly PDGF-AB and TGF-β1, than previous generations of PRF. 6 PDGF-AB and TGF-β1 attract osteoblasts and enhance their migration to bone remodeling sites. This chemotaxis is followed by osteogenic proliferation and differentiation. 24 25 Prior studies have examined the ability of the growth factors BMP-2, PDGF-AB, and TGF-β1 to induce migration in mesenchymal progenitor cells, obtaining data on the chemotactic index with no significant difference among BMP-2, PDGF, and TGF-β1. This study found that both PDGF and TGF-β increased primary osteoblast migration similarly to BMP-2. 26 The results of the observations showed a significant increase in osteoblast cell proliferation activity on days 1, 3, and 5. These findings align with those of a previous study which found that proliferation begins 24 hours after damage occurs, increasing until it reaches its maximum level on day 5. 27 The finding that osteoblast cell migration and proliferation peak after 5 days also correlates with the release of PDGF-AB and TGF-β1, which reach their maximum concentration after 3 to 7 days. Observation of osteoblast cell proliferation using fixed-angle and horizontal centrifugation on the first and third days revealed no significant difference. However, horizontal centrifugation showed a significant difference, indicating that C-PRF has a better capacity to increase proliferation in the horizontal group than in the fixed-angle group. This aligns with the 2020 findings of Fujioka-Kobayashi et al, who discovered that horizontal centrifugation substantially increases cell proliferation, migration, and differentiation activities, which are precursors to soft and hard tissue regeneration. 9 Additionally, a more recent study by Fujioka-Kobayashi et al showed that horizontal centrifugation significantly increased cell proliferation within 5 days compared to the control group. 16

A systematic review of 13 studies comparing horizontal and fixed-angle centrifugation concluded that 84.6% of researchers favored horizontal centrifugation, 15.4% reported no difference, and none favored fixed-angle centrifugation. 28 This suggests that horizontal centrifugation may lead to enhanced clinical outcomes when C-PRF is applied in various regenerative procedures. The results of the present study also support this suggestion. However, it is important to note that the small sample size and controlled variability in this study may have influenced the statistical significance of the data. Another limitation of this study is that the observation period was considerably short, since long-term observation may have affected the results. Additionally, the number of studies currently available on the regenerative potential of C-PRF application in clinical trials is limited. Therefore, further development and subsequent research are important to ensure the clinical relevance of these promising findings.

## Conclusion

Previous studies have shown evidence that the centrifugation method affects the regenerative potential of PRF, including growth factor release, cell migration, and proliferation. This *in vitro* study demonstrated that horizontal centrifugation is associated with higher TGF-β1 and PDGF-AB release, as well as enhanced osteoblast cell migration and proliferation, compared to fixed-angle centrifugation. Further *in vivo* and clinical studies with larger sample sizes, greater variability, longer observation periods, and more comprehensive experiments on regenerative potential are required to validate the significance of these findings.

## References

[1] Tattan M Periodontics the Complete Summary: Surgical Regenerative Therapy 1st ed. Batavia, IL Quintessence Publishing 2021 118 128

[2] Miron R J Choukroun J Wiley J Platelet Rich Fibrin in Regenerative Dentistry: Biological Background and Clinical Indications 1st ed. Hoboken, NJ Wiley 2017 4 5

[3] Rodriguez J C Bustillos M R Periodontics the Complete Summary: Tissue Engineering 1st ed. Batavia, IL Quintessence Publishing 2021 136 138

[4] Miron R J Estrin N E Ahmad P Thirty years of autologous platelet concentrates: from platelet-rich plasma to platelet-rich fibrin J Periodontal Res 2025 0 1 28 10.1111/jre.70013 40757985

[5] Miron R J Fujioka-Kobayashi M Hernandez M Injectable platelet rich fibrin (i-PRF): opportunities in regenerative dentistry? Clin Oral Investig 2017 21 08 2619 2627 10.1007/s00784-017-2063-9 28154995

[6] Miron R J Chai J Zhang P A novel method for harvesting concentrated platelet-rich fibrin (C-PRF) with a 10-fold increase in platelet and leukocyte yields Clin Oral Investig 2020 24 08 2819 2828 10.1007/s00784-019-03147-w 31788748

[7] Sari R Sukorini U Susilowati H Suryono S Comparative study on interdental papillae regeneration: leukocyte platelet-rich fibrin by-product versus hyaluronic acid injections in modified open gingival embrasure model Eur J Dent 2025(e-pub ahead of print) 10.1055/s-0045-1802948 PMC12890425 40311633

[8] Miron R J Chai J Zheng S Feng M Sculean A Zhang Y A novel method for evaluating and quantifying cell types in platelet rich fibrin and an introduction to horizontal centrifugation J Biomed Mater Res A 2019 107 10 2257 2271 31148358 10.1002/jbm.a.36734

[9] Fujioka-Kobayashi M Kono M Katagiri H Histological comparison of platelet rich fibrin clots prepared by fixed-angle versus horizontal centrifugation Platelets 2021 32 03 413 419 32306811 10.1080/09537104.2020.1754382

[10] Wang X Zhang Y Choukroun J Ghanaati S Miron R J Effects of an injectable platelet-rich fibrin on osteoblast behavior and bone tissue formation in comparison to platelet-rich plasma Platelets 2018 29 01 48 55 28351189 10.1080/09537104.2017.1293807

[11] Fujioka-Kobayashi M Katagiri H Kono M Improved growth factor delivery and cellular activity using concentrated platelet-rich fibrin (C-PRF) when compared with traditional injectable (i-PRF) protocols Clin Oral Investig 2020 24 12 4373 4383 10.1007/s00784-020-03303-7 32382929

[12] Caruana A Savina D Macedo J P Soares S C From platelet-rich plasma to advanced platelet-rich fibrin: biological achievements and clinical advances in modern surgery Eur J Dent 2019 13 02 280 286 31509878 10.1055/s-0039-1696585 PMC6777161

[13] Margono A Bagio D A Yulianto I Dewi S U Changes in migratory speed rate of human dental pulp stromal cells cultured in advanced platelet-rich fibrin Eur J Dent 2023 17 01 91 96 35436790 10.1055/s-0042-1743146 PMC9949916

[14] Staehlke S Rebl H Nebe B Phenotypic stability of the human MG-63 osteoblastic cell line at different passages Cell Biol Int 2019 43 01 22 32 30444078 10.1002/cbin.11073

[15] Liang C C Park A Y Guan J L In vitro scratch assay: a convenient and inexpensive method for analysis of cell migration in vitro Nat Protoc 2007 2 02 329 333 17406593 10.1038/nprot.2007.30

[16] Fujioka-Kobayashi M Schaller B Mourão C FAB Zhang Y Sculean A Miron R J Biological characterization of an injectable platelet-rich fibrin mixture consisting of autologous albumin gel and liquid platelet-rich fibrin (Alb-PRF) Platelets 2021 32 01 74 81 31959025 10.1080/09537104.2020.1717455

[17] Zwittnig K Kirnbauer B Jakse N Growth factor release within liquid and solid PRF J Clin Med 2022 11 17 5070 5070 36078998 10.3390/jcm11175070 PMC9456595

[18] Kargarpour Z Nasirzade J Panahipour L Miron R J Gruber R Relative centrifugal force (RCF; *G* -force) affects the distribution of TGF-β in PRF membranes produced using horizontal centrifugation Int J Mol Sci 2020 21 20 7629 33076376 10.3390/ijms21207629 PMC7589083

[19] Eppley B L Woodell J E Higgins J Platelet quantification and growth factor analysis from platelet-rich plasma: implications for wound healing Plast Reconstr Surg 2004 114 06 1502 1508 15509939 10.1097/01.prs.0000138251.07040.51

[20] Ferreira Sávio D S Silva L MPD Reis G GD Effects of platelet-rich fibrin produced by three centrifugation protocols on bone neoformation in defects created in rat calvaria Platelets 2023 34 01 2.228417E6 10.1080/09537104.2023.2228417 37409489

[21] Lourenço E S Alves G G de Lima Barbosa R Effects of rotor angle and time after centrifugation on the biological in vitro properties of platelet rich fibrin membranes J Biomed Mater Res B Appl Biomater 2021 109 01 60 68 32691512 10.1002/jbm.b.34680

[22] Gheno E Mourão C FAB Mello-Machado R C In vivo evaluation of the biocompatibility and biodegradation of a new denatured plasma membrane combined with liquid PRF (Alb-PRF) Platelets 2021 32 04 542 554 32531175 10.1080/09537104.2020.1775188

[23] Al-Maawi S Dohle E Kretschmer W Rutkowski J Sader R Ghanaati S A standardized *g* -force allows the preparation of similar platelet-rich fibrin qualities regardless of rotor angle Tissue Eng Part A 2022 28(7-8):353 365 34555949 10.1089/ten.TEA.2021.0113

[24] Mu Z He Q Xin L Effects of injectable platelet rich fibrin on bone remodeling in combination with DBBM in maxillary sinus elevation: a randomized preclinical study Am J Transl Res 2020 12 11 7312 7325 33312369 PMC7724338

[25] Gollapudi M Bajaj P Oza R R Injectable platelet-rich fibrin - a revolution in periodontal regeneration Cureus 2022 14 08 e28647 36196318 10.7759/cureus.28647 PMC9525133

[26] Fiedler J Röderer G Günther K P Brenner R E BMP-2, BMP-4, and PDGF-bb stimulate chemotactic migration of primary human mesenchymal progenitor cells J Cell Biochem 2002 87 03 305 312 12397612 10.1002/jcb.10309

[27] Azizi A Designing of artificial intelligence model-free controller based on output error to control wound healing process Biosens J 2017 6 01 1 19

[28] Farshidfar N Apaza Alccayhuaman K A Estrin N E Advantages of horizontal centrifugation of platelet-rich fibrin in regenerative medicine and dentistry Periodontol 2000 2025 00 1 52 10.1111/prd.12625 PMC13428090 40130760

